# Surgical resection of the primary tumor leads to prolonged survival in metastatic pancreatic neuroendocrine carcinoma

**DOI:** 10.1186/s12957-019-1597-5

**Published:** 2019-03-21

**Authors:** Tingting Feng, Wangxia Lv, Meiqin Yuan, Zhong Shi, Haijun Zhong, Sunbin Ling

**Affiliations:** 1 Department of Abdominal Medical Oncology, Zhejiang Cancer Hospital, Hangzhou, China; 2 Division of Hepatobiliary and Pancreatic Surgery, Department of Surgery, Collaborative Innovation Center for Diagnosis and Treatment of Infectious Diseases, the First Affiliated Hospital, Zhejiang University School of Medicine, 79 Qingchun Road, Hangzhou, 310003 China

**Keywords:** Pancreatic neuroendocrine carcinoma, Surveillance, Epidemiology, and End Results (SEER) database, Surgical therapy

## Abstract

**Background:**

Palliative resection of the primary tumor for metastatic pancreatic neuroendocrine carcinoma (pNEC) patients is not recommended because of the poor prognosis compared to that of patients with well-differentiated, lower grade tumors. However, the published data supporting this recommendation regarding pNEC are limited. In the present study, we assessed whether palliative primary tumor resection in stage IV pNEC patients affects survival and identified other factors that affect survival in these patients.

**Methods:**

We collected data from stage IV pNEC patients registered in the Surveillance, Epidemiology, and End Results (SEER) database between 1988 and 2014. Univariate and multivariate Cox regression analysis were used to compare overall survival (OS) and cancer-specific survival (CSS) of patients who did or did not undergo primary tumor resection.

**Results:**

We identified 350 patients with metastatic, poorly differentiated, and undifferentiated pNEC. A total of 14.3% (50/350) of patients underwent primary tumor resection. Multivariate Cox regression analysis showed that primary tumor resection provided a significant benefit for both OS and CSS in stage IV pNEC patients. Additionally, chemotherapy and the presence of the primary tumor in the pancreatic tail were independent positive prognostic factors for metastatic pNEC patients in the multivariate Cox regression analysis.

**Conclusions:**

The present study suggests that chemotherapy, location of the primary tumor in the pancreatic tail, and, most importantly, surgical removal of the primary tumor are associated with prolonged survival in stage IV pNEC patients.

**Electronic supplementary material:**

The online version of this article (10.1186/s12957-019-1597-5) contains supplementary material, which is available to authorized users.

## Introduction

Pancreatic neuroendocrine neoplasms (pNENs) are rare neoplasms derived from pancreatic neuroendocrine cells. pNENs have neuroendocrine markers and can secrete bioactive amine and peptide hormones. The reported incidence of pNEN is approximately 2.5–5 per 100,000 persons per year [1–4]. The World Health Organization (WHO) updated the naming and grading system for gastroentero-pancreatic neuroendocrine tumors (GEP-NET) in 2010. This system is based on the number of mitotic cells confirmed per 10 high-power fields (HPFs) and/or the Ki-67 labeling index. Grade 1 (G1) has < 2 mitotic cells/10 HPF and/or Ki-67 labeling index ≤ 2%, grade 2 (G2) has 2–20 mitotic cells/10 HPF and/or Ki-67 labeling index of 3–20%, and grade 3 (G3) has > 20 mitotic cells/10 HPF and/or Ki-67 labeling index > 20%. According to the histologic morphology and proliferation index, pNENs are divided into a well-differentiated subtype, pancreatic neuroendocrine tumors (pNETs) (G1, G2), and a poorly differentiated subtype, pancreatic neuroendocrine carcinomas (pNECs) (G3) [5]. In recent years, some pancreatic neuroendocrine tumors have been reported to have good morphologies and to be well differentiated, but their Ki-67 exceeded 20%, but it generally was not more than 60%, and this condition is currently referred to as a “high proliferative activity NET” or “well-differentiated G3 NET”. In 2017, WHO updated the grading of pancreatic neuroendocrine tumors and distinguished G3 pNET from G3 pNEC [6]. G3 NET has a significantly better prognosis than G3 NEC [7].

Currently, the AJCC staging of pNEN is based on primary tumor size, regional lymph node metastasis, and distant metastasis, while tumor differentiation is not considered [8]. As prognostic factors for patients with pNEN, there is controversy about the role of primary tumor size and regional lymph node metastasis [9]. Instead, distant metastasis is considered to be one of the strongest predictors of poor prognosis of patients with pNEN, and more than 60% of patients with pNEN have developed distant metastases at diagnosis [10]. Currently, platinum-based systemic chemotherapy is recommended as the first-line treatment for patients with pNEC, but this treatment achieves a median overall survival (OS) of only 5.8 to 12 months, and the 3-year OS rate reaches only 5–10% [11–13].

Given its highly aggressive biological behavior and poor prognosis, surgery for metastatic pNEC is not recommended, although published data on surgery for metastatic disease are scarce. Only two studies involving surgery for high-grade pNET and pNEC were referred to in the consensus guidelines of the European Neuroendocrine Tumor Society (ENETS) [14, 15]. Curative surgery is usually attempted in localized disease or for debulking or cytoreductive surgery, and metastatic resection is not recommended [16]. The consensus guidelines of the North American Neuroendocrine Tumor Society (NANETS) did not mention surgery as a treatment for metastatic high-grade pancreatic neuroendocrine carcinoma (hgpNEC) [17]. The European Society for Medical Oncology (ESMO) guidelines [18] and the National Comprehensive Cancer Network (NCCN) guidelines [19] do not recommend surgical treatment for patients with pNEC; however, it does not refer to relevant references as evidence for this statement.

The role of surgery in patients with hgpNEC is often controversial because patients are prone to recurrence. Neuroendocrine tumors with the grade G3 and distant metastasis are independent prognostic risk factors for patients with pNEN compared to G1/G2 stage and localized disease [20–22]. Therefore, we cannot deny the possible significance of surgical treatment for some metastatic pNEC patients. In addition, some reports discuss the positive effects of resection on survival [23, 24]. One study explored the role of operation in 119 patients with hgpNEC. The results showed that patients who underwent resection (*n* = 12) had a survival time of 29 months, and patients who underwent systemic chemotherapy alone had a survival time of only 13 months (*n* = 78) [25]. Moreover, some reports have described the positive effect of resection on survival [23, 24]. However, these studies are usually small series/case reports that lack a control group.

Given the limited evidence, this study was designed to assess the impact of primary tumor resection on the survival of patients with metastatic pNEC using the SEER database.

The SEER database is a program supported by the US National Cancer Institute. The database has collected data on the incidence, treatment, pathology, prognosis, and other information about cancer patients in the USA since 1973. Accessing the SEER database using the Site Recode ICD-O-3/WHO2008, we extracted all of the information about poorly differentiated and undifferentiated large-cell neuroendocrine carcinoma, small-cell carcinoma, and neuroendocrine carcinoma from the SEER database, which were matched with the newest definitions from the WHO 2010/2017 for pNEC.

## Materials and methods

### Study population and data sources

**Fig. 1:**
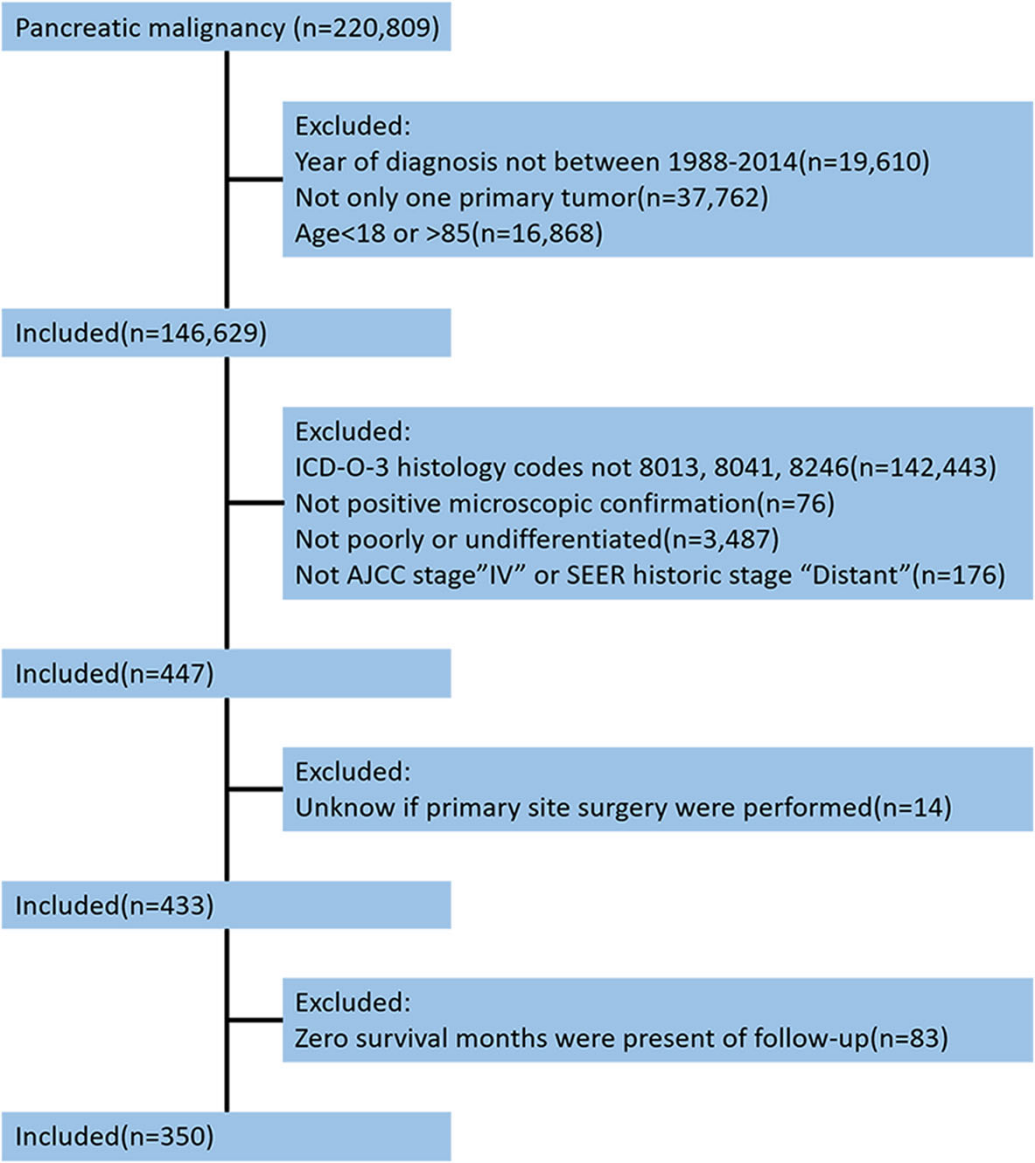
Exclusion criteria and study design

Information on cases was captured using SEER*Stat software (version 8.3.2—Nov 11, 2015; Cancer Statics Branch, NCI, Bethesda, MD) collected in the “Incidence SEER 18 Regs Custom data (with additional treatment field), Nov 2016 Sub (1973–2014 varying)” database. Site recode ICD-O-3/WHO 2008 “Pancreas” was used to identify pancreatic malignancy. Pancreatic malignancy patients diagnosed between the ages of 18 and 85 years inclusive from 1988 to 2014 were included. Patients were excluded if their pancreatic malignancy was not the only primary tumor; if the ICD-O-3 histology codes were not large-cell neuroendocrine carcinoma (8013), small-cell carcinoma (8041), or neuroendocrine carcinoma (8246); if they did not have positive microscopic diagnostic confirmation; if the grade was not poorly or undifferentiated; if the stage was not the American Joint Commission on Cancer (AJCC) stage “IV” or SEER historic stage “Distant”; if unknown primary tumor surgery was performed; and if zero survival months were present at follow-up (Fig. 1). The variables included were sex, race, age, grade, tumor size, tumor location, lymph node metastases, chemotherapy, year of diagnosis, site-specific surgery, resection of primary and distant sites, survival time, vital status, and cause-specific death.

### Statistical analysis

We performed statistical analysis using the Statistical package for Social Sciences (SPSS v.23 IBM Corporation, Armonk, NY). The chi-square test was used for group comparisons of categorical variables. Analysis of survival data was performed using the Kaplan–Meier method. Differences in survival between patient groups were compared using a log-rank test (Mantel–Cox). OS time was censored according to the vital status of patients. Cancer-specific survival (CSS) times are based on “cancer-specific death”. If the patient was living at the last follow-up, the survival time was censored. We calculated the median survival time and the associated 95% confidence interval of the patients. Univariate and multivariate analyses were performed using the Cox proportional hazard regression method. Categorical covariates were defined in univariate and multivariate Cox analysis, and the first category of each covariate was selected as the indicator for the reference category. All factors with *p* values less than 0.1 in the Cox univariate analysis were included in the multivariate analysis. Forward and backward stepwise selection methods were performed in the Cox multivariate analysis. All *p* values were two-tailed and were considered statistically significant if *p* < 0.05.

## Results

### Patient demographics and tumor characteristics

Among 350 patients with histologically diagnosed pNEC and distant metastases, 82.9% (290/350) were not treated by primary tumor resection, while 14.3% (50/350) underwent surgery to remove the primary tumor. Among these 50 patients, 50% (25/50) underwent both primary tumor and metastatic disease resection, and 50% (25/50) underwent only primary tumor resection. Compared to the nonsurgery group, more young patients (< 65 years of age, 76.0% vs 51.0%, *p* = 0.001, Table 1), more patients who had a body/tail tumor location (52.0% vs 27.3%, P<0.016, Table 1), and fewer patients who received chemotherapy (48.0% vs 65.7%, *P* = 0.017, Table 1) were found in the surgery group, but no differences were found for sex, race, grade, or tumor size (Table 1) between the two groups. There were more cases of positive lymph node metastasis in the primary tumor resection surgery group (74% vs 3%); however, the *p* value did not reach a statistical significance. A higher rate of lymph node sampling might be responsible for the greater number of lymph node-positive patients in the surgery group. Although the *p* value was less than 0.001 among all groups when comparing tumor size, there was no significance when the “unknown” group was removed. The median OS of patients with both primary tumor and distant disease resections was 19 months, while the median OS of the group with only primary site resection was 10 months. However, this difference did not reach statistical significance (*p* = 0.562).

**Table 1:** Baseline characteristics of the study groups

Variable	Group	Patient characteristics			
		Total *n* = 350	Resection of primary tumor *n* = 50	No resection of primary tumor *n* = 300	*p*
Sex					0.101
	Female	145 (41.4%)	26 (52.0%)	119 (39.7%)	
	Male	205 (58.6%)	24 (48.0%)	181 (60.3%)	
Race					0.726
	White	278 (79.4%)	39 (78.0%)	239 (79.7%)	
	Black	40 (11.4%)	5 (10.0%)	35 (11.7%)	
	Other	32 (9.1%)	6 (12.0%)	26 (8.7%)	
Age					0.001
	< 65 years	191 (54.6%)	38 (76.0%)	153 (51.0%)	
	≥ 65 years	159 (45.4%)	12 (24.0%)	147 (49.0%)	
Grade					0.284
	III	236 (67.4%)	37 (74.0%)	199 (66.3%)	
	IV	114 (32.6%)	13 (26.0%)	101 (33.7%)	
Tumor size					< 0.001^*^
	< 2 cm	4 (1.1%)	1 (2.0%)	3 (1.0%)	0.872^†^
	2–4 cm	96 (27.4%)	20 (40.0%)	76 (25.3%)	
	> 4 cm	152 (43.4%)	28 (56.0%)	124 (41.3%)	
	Unknown	98 (28.0%)	1 (2.0%)	97 (32.3%)	
Tumor location					< 0.001^*^
	Head/overlapping	177 (50.6%)	23 (46.0%)	154 (51.3%)	0.016^†^
	Body/tail	108 (30.9%)	26 (52.0%)	82 (27.3%)	
	Unknown	65 (18.6%)	1 (2.0%)	64 (21.3%)	
Lymph node metastases					< 0.001^*^
	No	14 (4.0%)	8 (16.0%)	6 (2.0%)	0.078^†^
	Yes	46 (13.1%)	37 (74.0%)	9 (3.0%)	
	Unknown	290 (82.9%)	5 (10.0%)	285 (95.0%)	
Chemotherapy					0.017
	No	129 (36.9%)	26 (52.0%)	103 (34.3%)	
	Yes	221 (63.1%)	24 (48.0%)	197 (65.7%)	
Year of diagnosis					0.099
	< 2010	212 (60.6%)	25 (50%)	187 (62.3%)	
	≥ 2010	138 (39.4%)	25 (50%)	113 (37.7%)	

^*^p value among all groups. ^†^*p* value among the groups except the “unknown” group

### Survival analysis

We calculated the median CSS times that corresponded to clinical factors of pNEC (Table 2). The median survival time of the primary site surgery group was longer than that of the nonsurgery group (12 months vs 8 months, Table 2). The reasons for not performing cancer-directed surgery included recommended but not performed and not recommended. The median survival times of the two nonsurgery groups (9 months and 7 months) were both significantly shorter than that of the surgery group (12 months, *p* < 0.001). Moreover, no significant difference was observed between the median survival times of the two nonsurgery groups. We also compared the subgroups of the surgery group, showing that patients with no primary site surgery but only distant metastasis resection achieved an 8-month median survival time, while patients who received primary site only or primary and distant site resection achieved a 10-month and 19-month median survival time, respectively. Moreover, the younger patients (less than 65 years old) achieved a longer survival than the older patients (9 months vs 6 months, *p* = 0.012, Table 2). Patients with a tumor located in the pancreatic tail (11 months) achieved a longer survival than those with tumors at other sites (*p* = 0.007, Table 2). Patients receiving chemotherapy achieved a longer survival time than patients who did not receive chemotherapy (10 months vs 4 months, *p* = 0.007, Table 2).

**Table 2:** Association of clinical and pathological variables with survival (cancer-specific survival)

Variable	Group	*N*	Median (minimum–maximum) (month)	95% CI	*p*
All		350	8 (1–138)	6.769–9.231	
Sex					0.309
	Female	145	9 (1–112)	7.605–10.395	
	Male	205	8 (1–138)	5.891–10.109	
Race					0.866
	White	278	9 (1–138)	7.411–10.589	
	Black	40	7 (1–36)	5.327–8.673	
	Other	32	8 (1–92)	2.824–13.176	
Age					0.012
	< 65y	191	9 (1–138)	7.592–10.408	
	≥ 65y	159	6 (1–106)	4.208–7.792	
Grade					0.177
	III	236	9 (1–138)	7.446–10.554	
	IV	114	8 (1–76)	6.462–9.538	
Tumor size					0.24
	< 2 cm	4	2 (2–28)	Undefined*	
	2–4 cm	96	9 (1–92)	6.880–11.120	
	> 4 cm	152	9 (1–138)	7.346–10.654	
Tumor location					0.007
	Head	146	8 (1–68)	6.399–9.601	
	Overlapping	31	9 (1–112)	7.121–10.879	
	Body	28	9 (1–106)	2.912–15.088	
	Tail	80	11 (1–138)	7.952–14.048	
Lymph node metastases					0.729
	No	14	9 (1–60)	0.000–37.636	
	Yes	46	11 (1–138)	7.032–14.968	
Chemotherapy					0.007
	No	129	4 (1–138)	2.763–5.237	
	Yes	221	10 (1–112)	8.841–11.159	
Year of diagnosis					0.087
	< 2010	212	8 (1–138)	6.698–9.302	
	≥ 2010	138	10 (1–50)	7.705–12.295	
Primary site surgery					< 0.001
	Surgery performed	50	12 (1–138)	2.079–21.921	
	Surgery not performed	300	8 (1–112)	6.594–9.406	
	Recommended but not performed	38	9 (1–76)	6.123–11.877	
	Not recommended and not performed	262	7 (1–112)	5.523–8.477	
Site specific surgery					0.39
	Local or partial pancreatectomy	24	26 (2–50)	11.541–40.459	
	Total pancreatectomy	4	8 (4–138)	0.000–95.547	
	Whipple	19	11 (1–68)	8.525–13.475	
	Unknown	3	9 (4–26)	0.998–17.002	
Primary site surgery					0.216
	Primary site only	25	10 (3–68)	3.129–16.871	
	Primary site and distant site	25	19 (1–138)	1.487–36.513	
	Distant site only	10	8 (1–112)	4.901–11.099	

^*^Too small sample size and too different survival data of the patients might lead to the undefinition of the corresponding 95% CI

**Fig. 2:**
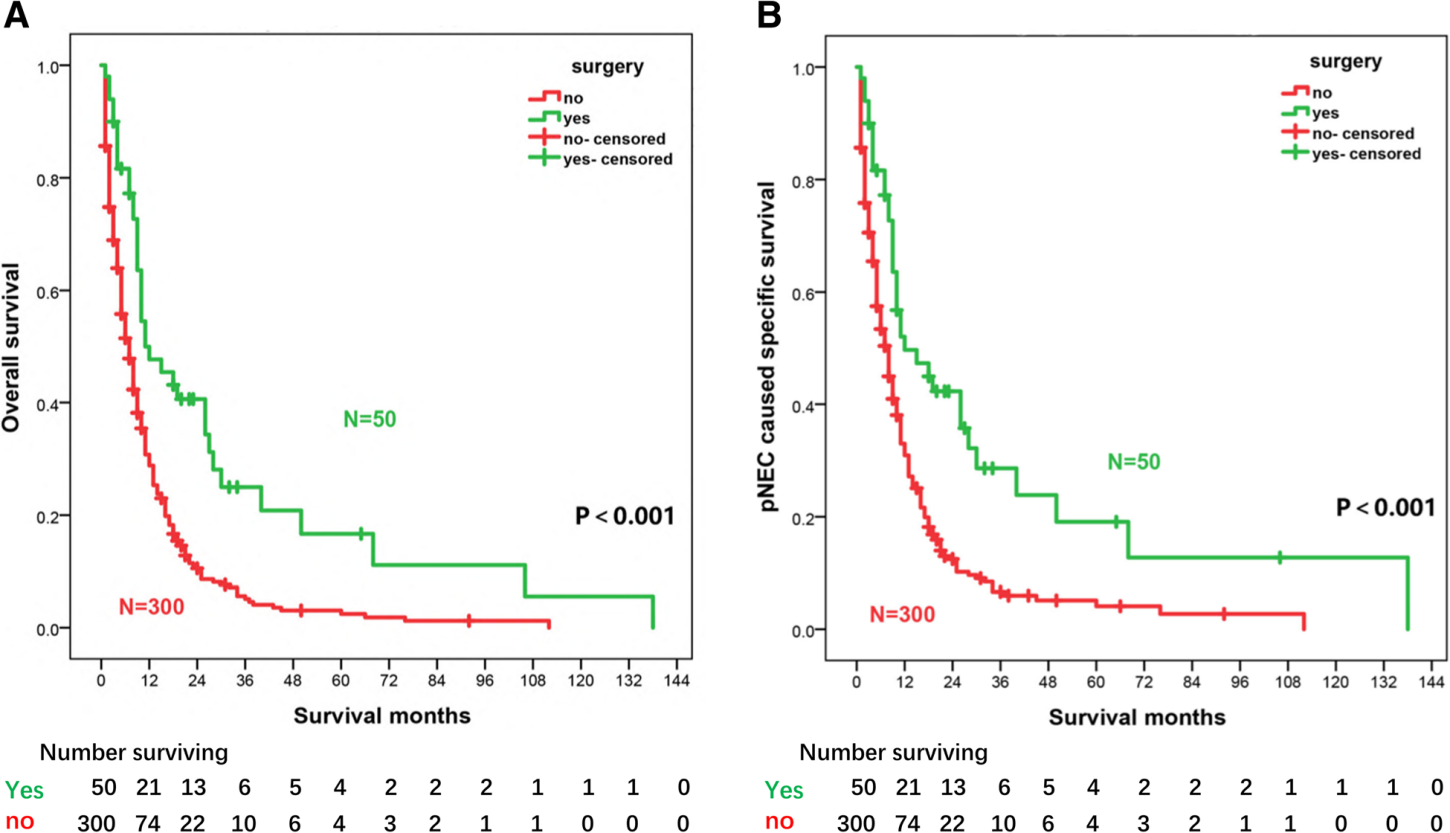
Kaplan-Meier curves for patients who did or did not undergo primary tumor resection. The overall survival (**a**) and cancer-specific survival (**b**) were displayed. The number of patients surviving in each group is given below each plot. *p* value was determined using a log-rank test

Figure 2a and b show the Kaplan–Meier curves of OS and CSS of the patients with or without primary site surgery performed. At each time point, both OS and CSS of the primary site surgery performed group were higher than those of the other group. Both univariate and multivariate Cox regression analyses showed that primary site resection was a prognostic protective factor of OS and CSS (OS, multivariate analysis, HR = 0.388, 95% CI, 0.269–0.560, *p* < 0.001; CSS, HR = 0.388, 95% CI, 0.262–0.576, *p* < 0.001, Table 3). Furthermore, chemotherapy and tumor location in the tail of the pancreas were also independent positive prognostic factors in Cox univariate and multivariate analyses (*p* < 0.001).

**Table 3:** Overall mortality and cancer-specific mortality in univariate and multivariable Cox regression analysis

	Overall mortality in Cox regression				Cancer-specific mortality in Cox regression			
	Univariable COX		Multivariable cox		Univariable cox		Multivariable cox	
	HR (95%CI)	*P*	HR (95%CI)	*P*	HR (95%CI)	*P*	HR (95%CI)	*P*
Sex								
Female(*n* = 145)	Reference	0.325			Reference	0.41		
Male(*n* = 205)	1.122 (0.892–1.413)				1.105 (0.871–1.402)			
Race								
White(*n* = 278)	Reference	.080			Reference	.182		
Black(*n* = 40)	1.357 (0.961–1.917)	0.083			1.333 (0.930–1.908)	0.117		
Other(*n* = 32)	0.773 (0.515–1.162)	0.216			0.847 (0.563–1.274)	0.425		
Age								
< 65 years (*n* = 191)	Reference	0.003			Reference	0.017		
≥ 65 years (*n* = 159)	1.413 (1.126–1.773)				1.333 (1.053–1.687)			
Grade								
III (*n* = 236)	Reference	0.106			Reference	0.197		
IV (*n* = 114)	1.223 (0.958–1.559)				1.181 (0.917–1.521)			
Tumor size								
< 2 cm (*n* = 4)	Reference	0.062			Reference	0.48		
2–4 cm (*n* = 96)	0.739 (0.270–2.025)	0.557			0.698 (0.254–1.915)	0.485		
> 4 cm (*n* = 152)	0.645 (0.238–1.750)	0.389			0.615 (0.226–1.670)	0.34		
Unknown (*n* = 98)	0.928 (0.340–2.531)	0.884						
Tumor location								
Head/overlapping/body (*n* = 205)	Reference	0.001	Reference	0.03	Reference	0.002	Reference	0.045
Tail (*n* = 80)	0.609 (0.456–0.815)	0.001	0.699(0.520–0.938)	0.017	0.600 (0.443–0.811)	0.001	0.685 (0.504–0.929)	0.015
Unknown(*n* = 65)	1.177 (0.871–1.590)	0.289	1.094(0.809–1.482)	0.559	1.069 (0.777–1.470)	0.682	0.999 (0.725–1.376)	0.995
Lymph node metastases								
No (*n* = 14)	Reference	0.001			Reference	0.003		
Yes (*n* = 46)	0.936 (0.479–1.971)	0.936			1.151 (0.529–2.501)	0.723		
Unknown(*n* = 290)	1.777 (0.941–3.353)	0.076			2.022 (0.997–4.103)	0.051		
Chemotherapy								
No (*n* = 129)	Reference	0.003	Reference	< 0.001	Reference	0.01	Reference	< 0.001
Yes (*n* = 221)	0.705 (0.558–0.890)		0.596(0.468–0.760)		0.726 (0.569–0.925)		0.624 (0.485–0.803)	
Year of diagnosis								
< 2010 (*n* = 212)	Reference	0.062			Reference	0.102		
≥ 2010 (*n* = 138)	0.791 (0.619–1.012)				0.810 (0.629–1.043)			
Primary surgery								
No (*n* = 300)	Reference	< 0.001	Reference	< 0.001	Reference	< 0.001	Reference	< 0.001
Yes (*n* = 50)	0.479 (0.337–0.682)		0.427(0.294–0.621)		0.487 (0.337–0.703)		0.440 (0.298–0.648)	

## Discussion

Recent studies have suggested that primary resection has positive prognostic significance for metastatic pNET [26–28], but studies about metastatic pNEC are limited. The SEER database accurately provides information on cancer statistics that have been collected for more than 30 years from the US population and is a good research tool for rare tumors such as pNEC. To the best of our knowledge, this is by far the largest study exploring the influence of primary tumor resection on the survival of metastatic pNEC patients.

In the present study, the median survival time of all patients was 8 months, similar to findings from previous research about pNEC [29–31]. At present, studies that analyze the prognosis of metastatic pNEC that are based on a large population are limited. This study analyzed in detail the correlation of median survival time with clinical factors and prognostic factors of pNEC that have concrete reference values.

By Cox regression analysis, our findings suggest that primary tumor resection is associated with longer OS and CSS in metastatic pNEC patients. Moreover, the OS or CSS rate of the patients with resection of the primary tumor is far better than that of patients in the nonresection group (Fig. 2), supporting the value of primary tumor site surgery in metastatic pNEC patients. Notably, further subgroup survival analysis for patients undergoing surgical treatment showed that the prognosis of patients with both primary and metastatic resection was significantly better than that of patients with only primary resection or only distant metastasis resection. Furthermore, we found that the majority of patients undergoing primary and metastatic site resection with detailed metastasis information may have only had liver metastases (Additional file 1: Table S1, to be published electronically). Accordingly, we propose the bold hypothesis that resection with curative intent, especially for liver metastasis patients, may improve prognosis more than palliative primary site surgical resection, although further research is needed to confirm this hypothesis. In fact, previous studies have reported that for patients with NET (G1/G2), primary tumor resection is beneficial to prolong OS [27, 32]. Moreover, Chakedis et al. found that removal of all metastatic disease in patients with metastatic NET was associated with the longest median survival (112.5 months) compared to that of debulking (89.2 months) or that of palliative resection (50.0 months; *p* < 0.001) [32]. Notably, Galleberg et al. evaluated the results of resection/radiofrequency ablation (RFA) with curative intent of liver metastases in patients with metastatic GEP-NEC. The median OS after resection/RFA of liver metastases was 35.9 months with a 5-year OS of 43% [33]. However, the survival time was shorter in our analysis. One obvious explanation for this is that the patients in the former study were mixed, with a variety of locations of neuroendocrine carcinoma in the digestive tract system, and this led to a difference in total median survival. Another possible explanation is that the location of the metastases and the extent of resection of the metastases could not be confirmed in our study. In addition, patients who underwent primary and metastatic resection may not have actually achieved radical resection. Lastly, the pNECs included in our study that were poorly differentiated or undifferentiated may have a worse prognosis than the high-grade NECs in their study. Conversely, a study reported that poorly differentiated colorectal NEC patients did not benefit from primary tumor resection [34]. Further exploration is needed of the causes of these differences. Consequently, methods of screening for metastatic pNEC patients who are good candidates for surgical intervention need further study.

Previous studies showed a median OS of 5.8 to 12 months of pNEC patients receiving chemotherapy [11–13], which is similar to the findings of the present study. Consistent with current guidelines for metastatic pNEC patients [16–18], our study also suggests that chemotherapy can improve outcomes of IV stage pNEC patients.

In addition, multivariable analyses showed that the pancreatic tail location of a primary tumor (compared to nontail locations) was a positive prognostic factor for survival. The effects of tumor location on the prognosis of pNEN are controversial. A previous study found that the prognosis of patients with a primary tumor in the body/tail was better than that of patients with a primary tumor in the head of the pancreas [35]. Tumors in the tail have a separate lymphatic drainage basin and are less likely to develop biliary, pancreatic, and visceral vessel compromise when compared to tumors in the head of the pancreas [27]. In this study, patients with a tumor in the pancreas body had a similar median survival time to the patients with a tumor in the head or overlapping regions of the pancreas. The small number of patients with a tumor in the pancreas body may be responsible for this difference. However, Tak et al. reported that tumors located in the body or tail of the pancreas were more likely to demonstrate a shorter progression-free survival (PFS) among pancreatic neuroendocrine tumors [36]. On the one hand, only pNET patients were included in that study. On the other hand, PFS cannot be considered equivalent to OS.

There are certain limitations to our study. This retrospective study cannot avoid the existence of selection bias. The SEER database does not provide information about tumor resection margin status, metastatic site information, disease burden, preoperational performance status, and other possible prognostic factors, such as the presence of comorbidities, complications, and additional life-prolonging therapy. Although a longer survival time was observed in patients after primary tumor resection in this retrospective study, further multicenter retrospective and prospective studies are needed to help the surgeons select ideal candidates, such as those with fewer metastases, smaller tumors, and better health to receive resections in the future.

## Conclusion

The result of this study showed that surgical removal of primary pNEC of the pancreases is associated with longer survival in patients with distant metastases. Additionally, chemotherapy and the presence of the primary tumor in the tail of the pancreas were independent positive prognostic factors for metastatic pNEC patients.

## Additional file

Additional file 1: **Table S1.** Bone/brain/liver/pulmonary metastases in 25 patients with primary and metastatic lesions resection. (DOCX 17 kb)

## Abbreviations

CSS: Cancer-specific survival
ENETS: European Neuroendocrine Tumor Society
ESMO: European Society for Medical Oncology
GEP-NET: Gastroentero-pancreatic neuroendocrine tumors
hgpNEC: High-grade pancreatic neuroendocrine carcinoma
HPFs: High-power fields
NANETS: North American Neuroendocrine Tumor Society
NCCN: National Comprehensive Cancer Network
NEC: Neuroendocrine carcinoma
OS: Overall survival
PFS: Progression-free survival
pNEC: Pancreatic neuroendocrine carcinoma
pNENs: Pancreatic neuroendocrine neoplasms
RFA: Resection/radiofrequency ablation
SEER: Surveillance, Epidemiology, and End Results
WHO: World Health Organization
