# Supplementary material for: Surgical resection of the primary tumor leads to prolonged survival in metastatic pancreatic neuroendocrine carcinoma
Source: World J Surg Oncol. 2019 Mar 21;17:54. doi: 10.1186/s12957-019-1597-5 (PMC6429809; doi:10.1186/s12957-019-1597-5)
Supplement: Supplementary file 1 — Table S1. Bone/brain/liver/pulmonary metastases in 25 patients with primary and metastatic lesions resection. (DOCX 17 kb) [file 12957_2019_1597_MOESM1_ESM.docx]

Additional file 1: **Table S1.** Bone/brain/liver/pulmonary metastases in 25 patients with primary and metastatic lesions resection.

| Patient Number | CS mets at DX-bone | CS mets at DX-brain | CS mets at DX-liver | CS mets at DX-lung |
| --- | --- | --- | --- | --- |
| 1 | 0 | 0 | 0 | 0 |
| 2 | 0 | 0 | 1 | 0 |
| 3 | 2 | 2 | 2 | 2 |
| 4 | 2 | 2 | 2 | 2 |
| 5 | 0 | 0 | 1 | 0 |
| 6 | 2 | 2 | 2 | 2 |
| 7 | 2 | 2 | 2 | 2 |
| 8 | 2 | 2 | 2 | 2 |
| 9 | 2 | 2 | 2 | 2 |
| 10 | 2 | 2 | 2 | 2 |
| 11 | 2 | 2 | 2 | 2 |
| 12 | 0 | 0 | 0 | 1 |
| 13 | 0 | 0 | 1 | 0 |
| 14 | 0 | 0 | 0 | 0 |
| 15 | 2 | 2 | 1 | 2 |
| 16 | 0 | 0 | 1 | 0 |
| 17 | 0 | 0 | 1 | 0 |
| 18 | 0 | 0 | 1 | 0 |
| 19 | 0 | 0 | 1 | 0 |
| 20 | 2 | 2 | 2 | 2 |
| 21 | 0 | 0 | 0 | 0 |
| 22 | 2 | 2 | 2 | 2 |
| 23 | 2 | 2 | 2 | 2 |
| 24 | 2 | 2 | 2 | 2 |
| 25 | 0 | 0 | 1 | 0 |

0：no 1：yes 2：unknown
